# Assessing Disparity Using Measures of Racial and Educational Isolation

**DOI:** 10.3390/ijerph18179384

**Published:** 2021-09-06

**Authors:** Mercedes A. Bravo, Man Chong Leong, Alan E. Gelfand, Marie Lynn Miranda

**Affiliations:** 1 Global Health Institute, Duke University, Durham, NC 27708, USA; mercedes.bravo@duke.edu; 2 Children’s Environmental Health Initiative, University of Notre Dame, South Bend, IN 46556, USA; mleong1@ufl.edu; 3 Department of Biostatistics, University of Florida, Gainesville, FL 32612, USA; 4 Department of Statistical Science, Duke University, Durham, NC 27708, USA; alan@stat.duke.edu; 5 Department of Applied and Computational Mathematics and Statistics, University of Notre Dame, Notre Dame, IN 46556, USA

**Keywords:** educational attainment isolation, racial isolation, local correlation, health disparities, spatial, birth outcomes

## Abstract

We develop a local, spatial measure of educational isolation (EI) and characterize the relationship between EI and our previously developed measure of racial isolation (RI). EI measures the extent to which non-college educated individuals are exposed primarily to other non-college educated individuals. To characterize how the RI-EI relationship varies across space, we propose a novel measure of local correlation. Using birth records from the State of Michigan (2005–2012), we estimate associations between RI, EI, and birth outcomes. EI was lower in urban communities and higher in rural communities, while RI was highest in urban areas and parts of the southeastern United States (US). We observed greater heterogeneity in EI in low RI tracts, especially in non-urban tracts; residents of high RI tracts are likely to be both educationally and racially isolated. Associations were also observed between RI, EI, and gestational length (weeks) and preterm birth (PTB). For example, moving from the lowest to the highest quintile of RI was associated with a 1.11 (1.07, 1.15) and 1.16 (1.10, 1.22) increase in odds of PTB among NHB and NHW women, respectively. Moving from the lowest to the highest quintile of EI was associated with a 1.07 (1.02, 1.12) and 1.03 (1.00, 1.05) increase in odds of PTB among NHB and NHW women, respectively. This work provides three tools (RI, EI, and the local correlation measure) to researchers and policymakers interested in how residential isolation shapes disparate outcomes.

## 1. Introduction

Health disparities research often focuses on race, which is non-modifiable, as a driver of differential outcomes. In this paper, we attempt to shift the conversation to the *experience of racial minorities*, which is modifiable, as a key driver of health outcomes, and provide tools to other public health researchers and policymakers. Our particular focus within the experience of racial minorities is on their relative racial and educational isolation.

Racial residential segregation (RRS) of blacks refers to the geographic separation of blacks from other racial/ethnic groups [1]. Through the concentration of poverty and poor physical and social environments, RRS results in distinctive environments that may underlie racial disparities in health outcomes [1]. Research has linked RRS with infant and adult mortality [2,3], poor pregnancy outcomes [4,5], type 2 diabetes [6], hypertension [7,8], and poor cardiovascular health [9]. Despite legal measures to address segregation, segregation and its consequences persist in the United States [10].

RRS is a multidimensional phenomenon, which has been characterized by five distinct dimensions: evenness, isolation, concentration, centralization, and clustering [11]. Racial isolation (RI) is defined as the extent to which minorities are exposed to majority group members by sharing a residential neighborhood. Importantly, it is not the exposure to other racial groups that is posited to improve outcomes, but rather the accumulation of social and environmental stressors in isolated neighborhoods that is posited to lead to poorer outcomes.

RRS measures have commonly been conceived at the Metropolitan Statistical Area (MSA) [12,13,14], although recently, researchers have started considering smaller analytical units, including next-door neighbors [15]. A review article of segregation and health identified two ways in which studies of RRS and health have conceptualized segregation [16]: (1) a formal measure of geographical segregation of racial groups with indices reflecting either exposure/isolation, evenness, concentration, centralization, or clustering [11]; and (2) a proxy measure, e.g., Black racial composition, % non-Hispanic Black (NHB). Prior assessments of segregation and health have found it conceptually problematic to conflate formal vs. proxy measures [17]. Of studies using a formal measure of segregation (as we do here), 33 studies measured segregation at the MSA or city level; 3 studies measured segregation at the county level; 1 study was at the state level; and no studies were conducted at the census tract level, although one study examined the proxy measure, racial composition, at the tract level. For example, MSA and city-level measures can assess whether Detroit is more segregated than Atlanta but not whether a particular neighborhood or other small area is more segregated than another within the same larger geographic region nor do they allow an assessment of whether a neighborhood or other small area has become more or less segregated over time.

Furthermore, of the studies examined in the review article described above, 18 used a measure of evenness to assess RRS, compared to 12 studies using exposure/isolation, 2 studies using concentration and clustering, and 1 study using centralization [16]. There is an extensive literature on residential segregation and its causes [11,18,19,20,21,22] as well as methodological issues (e.g., formal vs. proxy measures of segregation, uses, and advantages of different types of indices such as exposure/isolation vs. dissimilarity) [16,17,23,24,25]. In this literature, some researchers argued that compared to the commonly employed dimension of evenness (dissimilarity), exposure/isolation may be more closely linked to health by serving as a proxy for the concentration of multiple disadvantages into a single ecological space [17,26]. With this literature as context, we developed a local, spatial measure of RI in previous work [4]. The local RI index, which we implement here at the census tract level, may be more directly linked to individual health than measures of segregation at the city or metropolitan area levels [4]. Unlike aspatial measures of segregation, our spatial index accounts for relationships among nearby census tracts.

In this paper, we apply the isolation concept to educational attainment. Using the same methodological approach as that used for RI, we develop a measure of educational isolation (EI) that evaluates to what extent individuals without a college degree are exposed only to other individuals without a college degree. Educational attainment has been consistently linked to health outcomes [27,28,29,30,31,32].The current literature around education and segregation is more focused on racial/ethnic and/or socioeconomic segregation in public schools [33,34,35], which is driven, at least in part, by racial/ethnic and socioeconomic residential segregation [36]. School segregation and poverty, as well as its drivers and consequences, is an important topic [37] because it is strongly associated with the magnitude of achievement gaps in early childhood and with the rate at which gaps grow over time [38]. Yet there is little information on residential segregation by educational attainment, which we examine here. Our EI index allows researchers to assess the importance of access to those with higher educational attainment along with the more traditional individual-level educational attainment measures. This approach extends the arguments laid out by Charles Putnam in Chapter 4 of *Our Kids* [39].

Furthermore, an ongoing debate in the US (and elsewhere) revolves around whether race serves as a proxy for socioeconomic status or whether race and socioeconomic status individually and jointly drive disparate outcomes [40,41]. Thus, this paper also explores the relationship between EI and RI, given that educational attainment is one important component of socioeconomic status. This dual approach is especially important given the enduring legacy of racism in the US. To evaluate the relationship between RI and EI, we propose a novel measure of local correlation. This novel measure assesses if and how the correlation between EI and RI varies across space. Widely used measures of global correlation (e.g., Pearson correlation coefficient) provide an area-wide measure of correlation which, while useful, does not indicate whether and to what degree the relationship between the variables of interest is uniform versus heterogeneous over the study area. Using the local measure of correlation, we evaluate the presence and nature of spatial variability in the relationship between RI and EI. Critically, this measure of local correlation can be applied to any combination of variables and will be useful to researchers or policymakers examining relationships between variables reported at an areal unit (e.g., poverty, crime, environmental exposures such as air pollution, health outcomes, reported at block, block group, census tract, zip code, county, neighborhood).

In this work, we (1) develop a local, spatial measure of EI; (2) calculate local, spatial measures of RI and EI, at the census tract level, across the continental US; (3) characterize the relationship between EI and RI using a standard measure of global correlation and a novel measure of local correlation, and by urbanicity and region within the US; and (4) assess the relationship between RI, EI, and pregnancy outcomes using detailed birth records from the State of Michigan. This work provides new tools for health disparity and policy researchers as well as actionable information for policymakers.

## 2. Materials and Methods

### 2.1. Study Area

We focus on the 48 states of the continental US, assessing RI and EI in the 72,706 census tracts designated in the 2010 U.S. Census. Of these tracts, 496 had no population and were excluded from the analysis, resulting in an analysis dataset of 72,210 census tracts with a mean (standard deviation) and median population size of 4257 (1955) and 4012, respectively. The population of census tracts ranged from a minimum of 1 to a maximum of 37,452 individuals.

Of 327 million people in the US, approximately 77% are non-Hispanic white (NHW), 13% are NHB, and 31% have a college degree. These statistics mask substantial heterogeneity in racial/ethnic composition and educational attainment across the country. For example, approximately 38% of the population in Mississippi is NHB, compared to just under 1% in Idaho. In West Virginia; meanwhile, fewer than 20% of adults have a college degree, compared to 42% in Massachusetts [42].

### 2.2. Data

We obtain count data on race/ethnicity and educational attainment at the tract level from the 2010 Census and American Community Survey (ACS) [42].

Urbanicity is determined using primary and secondary rural–urban commuting area (RUCA) codes, which are assigned by the US Department of Agriculture based on population density, urbanization, and the size and direction of daily commuting flows [43]. We use RUCA codes to classify tracts into urban, suburban, and rural categories. RUCA codes are based on the 2010 decennial census data and the 2006–2010 ACS data (see Table S1).

### 2.3. Isolation Measures

#### 2.3.1. Racial Isolation

We calculate our previously developed local, spatial measure of RI of self-identifying NHB individuals (compared with all other racial/ethnic groups, including Hispanics) in each tract for the continental US [4]:

(1) $$RI_{im}=\left(\underset{j\in \partial_{i}}{\sum }w_{ij}T_{jm}\right)/\left(\underset{j\in \partial_{i}}{\sum }w_{ij}T_{j}\right).$$

In Equation (1), $\partial_{i}$ denotes the set of index unit (i) and its neighbors (i.e., tracts that are adjacent to the index tract). Given M mutually exclusive racial subgroups, m indexes the subgroups of M (e.g., NHB). $T_{i}\;$ denotes the total population in region i, and $T_{im}$ denotes the population of subgroup m in region i. $\left(w_{ij}\right)$ denotes a n×n first-order adjacency matrix, where *n* is the number of census tracts in the study area. First-order adjacency means that the entries in the matrix, $w_{ij}$, are set to 1 if a boundary is shared by region i and region j, and 0 otherwise. Entries of the main diagonal (since $i\in \partial_{i},\;w_{ij}=w_{ii}$ when j=i) of $\left(w_{ij}\right)$ are set to 1.5, such that the weight of the index unit, i, is larger than the weights assigned to adjacent tracts. Since we are more interested in the spatial patterns rather than the aspatial patterns, $w_{ii}$ should not be set to too high. For neighbors of any index unit i with 0 population, the corresponding $T_{jm}$ and $T_{j}$ are 0, so that the value of $RI_{im}$, the RI index of unit i for subgroup m, would not be affected. We note that in calculating spatial indices, edge tracts (e.g., tracts along a coastline or bordering Canada or Mexico) may have few neighboring tracts; index values in these tracts may be unstable.

The resulting RI index ranges from 0 to 1. A neighborhood environment that is nearly all non-NHB will have an RI value that is close to 0. In contrast, a neighborhood environment that is nearly all NHB will have an RI value that is close to 1 [44].

#### 2.3.2. Educational Isolation

We develop an analogous local, spatial measure of EI, assessing likelihood of living in the same neighborhood of individuals without a college degree to those with a college degree. We calculate tract-level EI scores by accounting for the population composition in the index tract along with adjacent tracts.

(2) $$EI_{im}=\left(\underset{j\in \partial_{i}}{\sum }w_{ij}T_{jm}\right)/\left(\underset{j\in \partial_{i}}{\sum }w_{ij}T_{j}\right)$$

In Equation (2), the value of $EI_{im}$, the educational isolation index of unit i for subgroup m, is calculated in the same way as $RI_{im}$, except m indexes mutually exclusive subgroups of educational attainment categories (e.g., individuals with a four-year college degree, individuals without a four-year college degree). Note that the right-hand sides of Equations (1) and (2) are identical. These equations only differ in terms of how subgroup *m* is defined. The EI index ranges from 0 to 1. A neighborhood environment that is nearly all college educated will have an educational isolation value that is close to 0. In contrast, a neighborhood environment that is nearly all non-college educated will have an educational isolation value that is close to 1 [45].

The resulting RI or EI value represents a weighted average proportion of the population that is NHB, in the case of RI, or that is not college educated, in the case of EI. As presented here, RI and EI assign greater weight to the index census tract (i.e., the census tract for which the value is being calculated) and somewhat less weight to census tracts that are adjacent to the index tract.

### 2.4. Relationships between RI and EI

#### 2.4.1. Measures of Correlation

We calculate global and local measures of correlation between RI and EI. The Pearson correlation coefficient is used to measure correlations at the national level (Equation (3)).

(3) $$R=\frac{\sum_{i}(Y_{i}-\bar{Y})\left(X_{i}-\bar{X}\right)}{\sqrt{\sum_{i}{\left(Y_{i}-\bar{Y}\right)}^{2}\sum_{i}{\left(X_{i}-\bar{X}\right)}^{2}}}$$

In Equation (3), $X_{i}$ and $Y_{i}$ denote the value of variables X (e.g., RI) and Y (e.g., EI) for areal unit (census tract) i, respectively. $\bar{X}$ and $\bar{Y}$ denote the average of the Xs and Ys across the study area. We present this widely known representation of the Pearson correlation coefficient to provide ease of interpretability of the local spatial measure of correlation presented below.

The Pearson correlation coefficient is aspatial and fails to capture how the relationship between RI and EI may vary across space. Thus, we propose a local measure of correlation (Equation (4)).

(4) $$R_{i}=\frac{\sum_{j\in \partial_{i}}w_{ij}\left(Y_{j}-{\bar{Y}}_{i}\right)\left(X_{j}-{\bar{X}}_{i}\right)}{\sqrt{\sum_{j\in \partial_{i}}w_{ij}{\left(Y_{j}-{\bar{Y}}_{i}\right)}^{2}\sum_{j\in \partial_{i}}w_{ij}{\left(X_{j}-{\bar{X}}_{i}\right)}^{2}}}$$

Similar to Equation (1), in Equation (4), $\partial_{i}$ denotes the set of index unit (i) and its neighbors. Here, we choose to include first-order neighbors (i.e., tracts adjacent to the index tract) and second-order neighbors (i.e., tracts adjacent to first-order neighbors of the index tract) in $\partial_{i}$. If we only include first-order neighbors in $\partial_{i}$, for each unit i, with only a few of the $w_{ij}\neq 0$, the local correlations are computed using few values (neighbors), producing a potentially unstable result. ${\bar{X}}_{i}$ and ${\bar{Y}}_{i}$ denote the weighted average of the Xs and Ys over unit *i* and its neighbors.

Given $\left(w_{ij}\right)$ (n×n matrix) denoting the matrix that we use to calculate $R_{i}$, entries $w_{ij}$ are set to a positive value if, for a given unit i, unit j $\in \partial_{i}$, i.e., is a first or second-order neighbor of unit i, or if *i* = *j*. Note that the $w_{ij}$ term allows us to specify different weights for different neighbors. Intuitively, the index unit (census tract) i has the greatest influence on itself, so $w_{ii}$ is set to a larger value ($w_{ii}=1.5$); first-order neighbors of unit i have less influence on unit i than unit i itself, so the corresponding $w_{ij}$ are set smaller than $w_{ii}$ ($w_{ij}=1$). Second-order neighbors of unit i have less influence on tract i than the first-order neighbors; so the corresponding $w_{ij}$ are set smaller than the $w_{ij}$ of the first-order neighbors ($w_{ij}=0.5$). $w_{ii}$ and $w_{ij}$ can be adjusted according to spatial scale, research question of interest, or the interactions between and within areal units.

This local measure of correlation describes the relationship between the neighborhood environment of an index tract with respect to two different variables. In this case, the neighborhood environment is defined as tracts that are adjacent to the index tract (first-order neighbors) as well as tracts adjacent to the first-order neighbors of the index tract (second-order neighbors). In calculating the local measure of correlation, greater weight is assigned to first-order neighbor tracts, and less weight is assigned to second-order neighbor tracts. The resulting local correlation measure ranges from −1 to 1. A local correlation value of 0 indicates no relationship between variable values—in this case, RI and EI—in the neighborhood environment (defined as first and second-order neighbors). A local correlation value near 1 indicates that RI and EI in the defined neighborhood environment are strongly positively correlated with one another. For example, this would occur when the RI values of a given neighborhood are similar to the EI values of the neighborhood. A local correlation value approaching −1 indicates that RI and EI values in the neighborhood are strongly negatively correlated with one another. This indicates that RI and EI values in the neighborhood environment are very different from one another. Thus, the measure of local correlation provides information regarding whether RI and EI values in a neighborhood are similar to ($R_{i}\to 1)\;$, unrelated to ($R_{i}=0)$, or different from ($R_{i}\to -1)\;$ one another. In this work, we calculate local correlation between RI and EI, but this measure can be applied to other variables as well. 

#### 2.4.2. Urbanicity

Using RUCA codes to assign urbanicity [43], we examine the relationship between RI and EI separately for urban, suburban, and rural tracts. The RUCA code classification scheme is provided in the Supplemental Material (SM), Table S1.

### 2.5. Public Health Relevance: RI, EI, and Pregnancy Outcomes

We examined associations between RI, EI, and gestational age and preterm birth (PTB) in the State of Michigan. We obtained detailed birth records from the Michigan Department of Community Health Vital Records, which included 1,608,537 births between 2000 and 2012. We restricted the dataset to births between 1 January 2005 and 31 December 2012 (*n* = 954,455) that were geocoded to a 2010 census tract (*n* = 942,422). Then, we restricted the dataset to singleton births (i.e., no plural births), without known congenital anomalies, birthweight >400 g, clinical estimate of gestational age 24–42 weeks, birth-order <4, maternal age range 15–44 years (y), and maternal race of NHB or NHW (*n* = 822,290). We also removed 14,299 records with missing values, such as maternal educational attainment, age, tobacco use during pregnancy, marital status, and census tract RI or EI. The final analysis dataset included 807,991 infants.

Infants were assigned RI and EI values based on the maternal census tract of residence at time of birth (using RI and EI calculated from 2010 Census data). The birth outcomes of interest were gestational age in weeks and PTB (0/1), defined as a birth occurring prior to 37 weeks gestation, based on the clinical estimate of gestational age. Race-stratified, multi-level models of birth outcomes were adjusted for individual-level characteristics, including maternal age, educational attainment, marital status, tobacco use during pregnancy, and infant sex, as well as census tract level RI and EI. Census tract of maternal residence at time of birth was included as random effects to account for potential unobserved heterogeneity. As a sensitivity analysis, we also fit models that included percentage NHB instead of RI and percentage non-college educated instead of EI to assess if and how these associations differed from one another.

This research was approved by the Institutional Review Boards at University of Notre Dame and the Michigan Department of Health and Human Services.

## 3. Results

### 3.1. Summary Statistics

**Figure 1:**
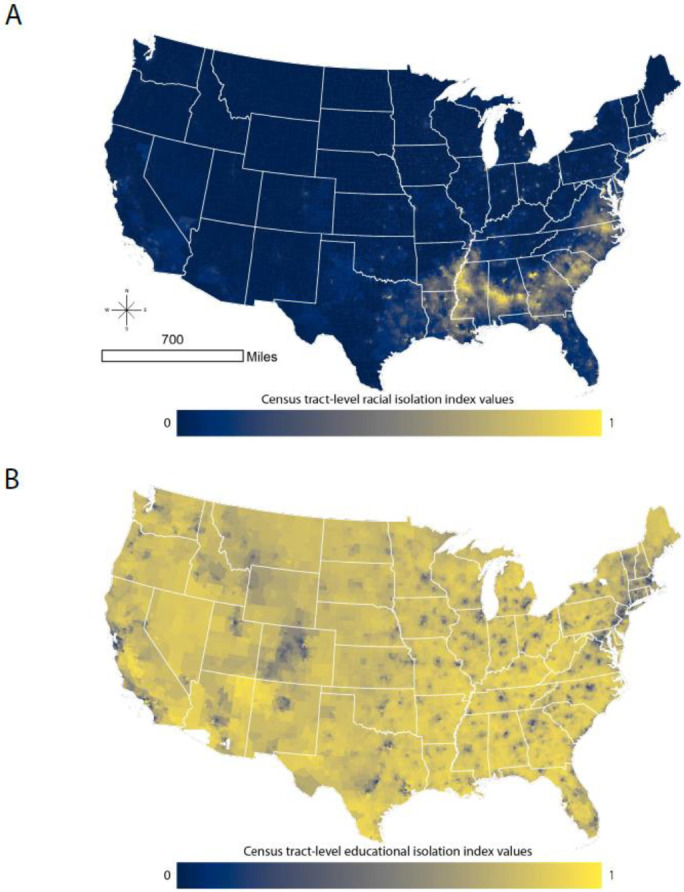
Census tract-level racial isolation index values (Panel **A**) and educational isolation index values (Panel **B**). Map projection: USA Contiguous Albers Equal Area Conic. RI ranges from 0 to 1. A value of 0 indicates that the neighborhood environment (defined as the index tract and tracts adjacent to the index tract) is entirely composed of non-NHB individuals. A value of 1 indicates that the neighborhood environment is entirely composed of NHB individuals. EI also ranges from 0 to 1. A value of 0 indicates that the neighborhood environment is entirely composed of individuals with a four-year college degree. A value of 1 indicates that the neighborhood environment is entirely composed of non-college educated individuals.

Figure 1A,B map the distribution of RI and EI across the continental US. To aid with interpretation, an RI value of 1 indicates that the neighborhood environment (defined as the index tract and tracts adjacent to the index tract) is entirely composed of NHB individuals, i.e., there is complete isolation of NHB persons from non-NHB persons. An RI value of 0 indicates that the neighborhood environment is entirely composed of non-NHB individuals, i.e., there is complete isolation of non-NHB persons from NHB persons. Similarly, an EI value of 1 indicates that the neighborhood environment is entirely composed of individuals without a four-year college degree, i.e., there is complete isolation of non-college educated persons from college educated persons. An EI value of 0 indicates that the neighborhood environment is entirely composed of college educated individuals, i.e., there is complete isolation of college educated persons from non-college educated persons. Tract-level RI ranges from 0 to 0.98. Mean (standard deviation) and median tract-level RI values are 0.13 (0.19) and 0.05, respectively. Tract-level EI values range from 0.09 to 1. The mean (standard deviation) and median tract-level EI values are 0.72 (0.16) and 0.76, respectively. Clearly, the patterns of EI and RI differ substantially across the US. RI is relatively low across much of the continental US, but it is high across the southeast and in urban areas along and east of the Mississippi River and in large western urban areas. EI is more likely to be lower in urban areas and higher in rural areas.

**Figure 2:**
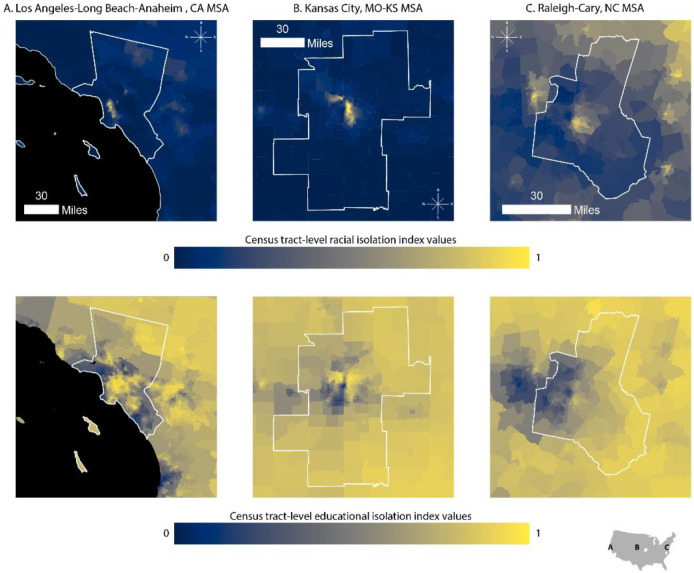
Census-tract level RI and EI in three MSAs: Los Angeles-Long Beach-Anaheim (Panel **A**); Kansas City (Panel **B**); and Raleigh-Cary (Panel **C**). RI ranges from 0 to 1. A value of 0 indicates that the neighborhood environment (defined as the index tract and tracts adjacent to the index tract) is entirely composed of non-NHB individuals. A value of 1 indicates that the neighborhood environment is entirely composed of NHB individuals. EI also ranges from 0 to 1. A value of 0 indicates that the neighborhood environment is entirely composed of individuals with a four-year college degree. A value of 1 indicates that the neighborhood environment is entirely composed of non-college educated individuals. Each MSA is projected to the appropriate projected coordinate system for its location in space, specifically: (Panel **A**) NAD 1983 State Plane California V FIPS 0405 Feet; (Panel **B**) North American Datum (NAD) 1983 Universal Transverse Mercator (UTM) Zone 14N; (Panel **C**) NAD 1983 State Plane North Carolina FIPS 3200 Feet.

While Figure 1 A,B present the variability in RI and EI across the country, considerable local variability is lost in these displays. In Figure 2, we zoom in on the Kansas City, Los Angeles-Long Beach-Anaheim, and Raleigh-Cary MSAs to demonstrate within-MSA variability in both EI and RI. We selected these cities because they come from distinct regions of the US, differ in terms of economy, population size, and population demographics, and show variable patterns in RI, EI, and the correlation between the two.

**Figure 3:**
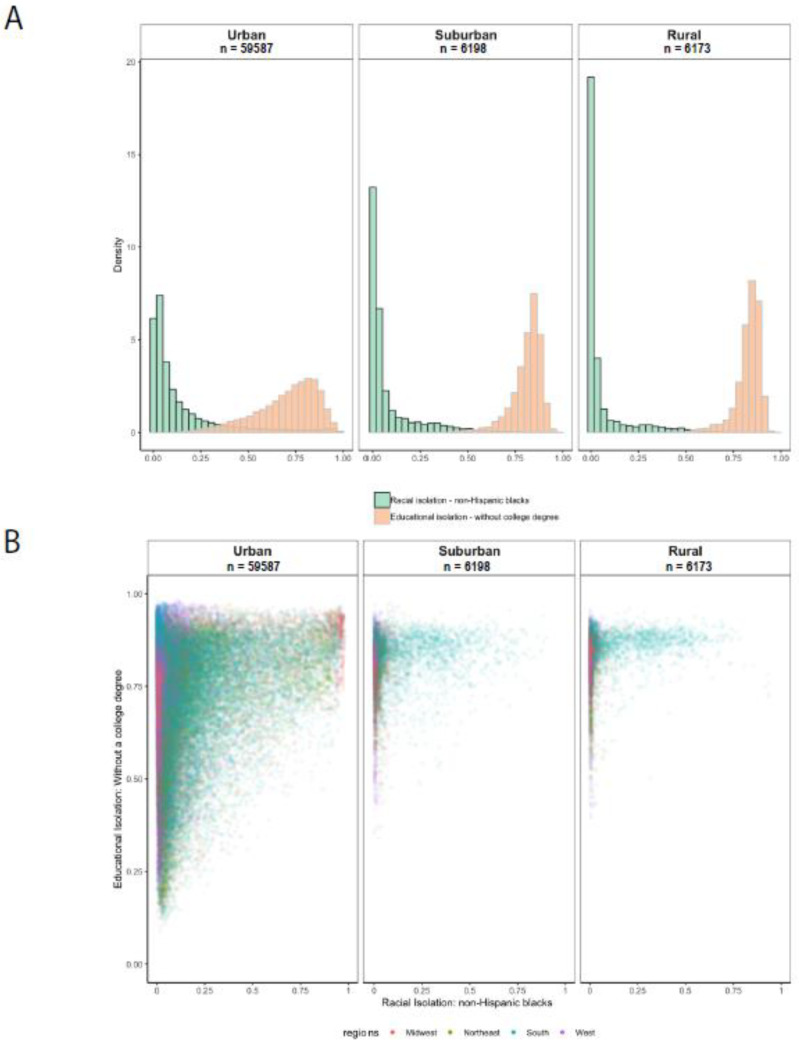
Distribution of RI and EI for the continental US in urban, suburban, and rural census tracts (Panel **A**) and plot of EI index values versus RI index values for urban, suburban, and rural census tracts (Panel **B**).

Figure 3, Panel A shows histograms of the distribution of RI and EI for urban, suburban, and rural census tracts. The RI distribution is right-skewed for urban, suburban, and rural census tracts. Compared to urban census tracts, a higher proportion of rural census tracts have very low RI values, which is likely because many rural census tracts are predominantly populated by non-Hispanic whites [46]. 

In contrast to RI, the EI distribution is left-skewed. Compared to urban census tracts, a larger proportion of suburban and rural census tracts have high values of EI (non-college-educated have little interaction with college-educated), indicating that rural and suburban tracts tend to have a greater concentration of individuals without a college degree who are only living in proximity to other individuals without a college degree [47]. The left-skewed EI distribution for suburban tracts may be related to how urbanicity is defined using rural–urban commuting area codes, which are determined using information on population density, urbanization, and the size and direction of daily commuting flows. 

### 3.2. Relationship between EI and RI

The global correlation between EI and RI is 0.21 for the continental US. This value belies the substantial variation across the country. Plots of EI vs. RI for urban, suburban, and rural census tracts are provided in Figure 3, Panel B. We observe greater heterogeneity in EI values in tracts with low levels of RI than in tracts with high levels of RI: across all degrees of urbanicity, tracts with RI values near zero exhibit a wider range of EI values than tracts with high RI levels. This pattern is most pronounced for suburban and rural tracts. Residents of a tract with low RI—especially in suburban or rural areas—may or may not experience high EI, while in a tract with high RI, residents are more likely to be both educationally and racially isolated. Only the south has tracts with a range of RI values in urban, suburban, and rural tracts. That is, in other regions of the US, there are few suburban or rural tracts—or none—with medium or high levels of RI. 

**Figure 4:**
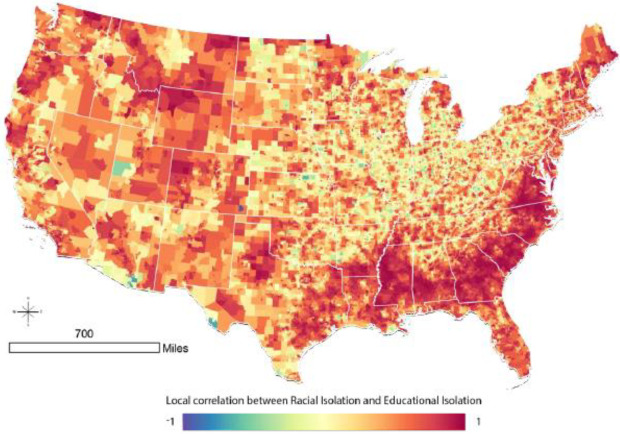
Local correlation between racial isolation and educational isolation.

To understand the relationship between EI and RI at the local level, we calculate and map tract-level local correlation coefficients for census tracts across the continental US (Figure 4). In much of the Deep South, and extending into non-Appalachian areas of North Carolina and Virginia, there is a strong correlation between RI and EI. The South has many tracts with high levels of RI (Figure 1, Panel B), and the patterning of local correlation in Figure 4 indicates that many of the highly racially isolated tracts in the south are also educationally isolated.

High correlations between EI and RI are observed elsewhere. However, these differ fundamentally from the south, because tracts in these regions more often have low levels of RI (Figure 1, Panel A). Therefore, a high correlation between EI and RI in areas outside the south may reflect low levels of both RI and EI. 

**Figure 5:**
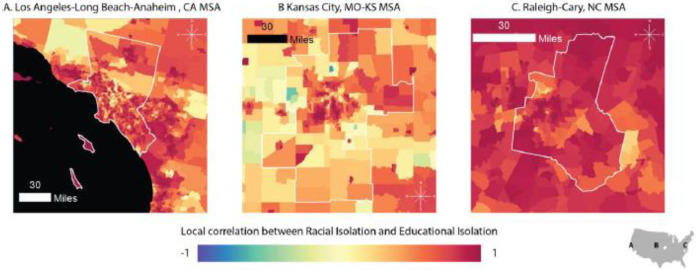
Census-tract level local correlation between racial isolation and educational isolation in three MSAs: Los Angeles–Long Beach–Anaheim (Panel **A**); Kansas City (Panel **B**); and Raleigh–Cary (Panel **C**). Each MSA is projected to the appropriate projected coordinate system for its location in space, specifically: Panel **A**: NAD 1983 State Plane California V FIPS 0405 Feet; Panel **B**: North American Datum (NAD) 1983 Universal Transverse Mercator (UTM) Zone 14N; Panel **C**: NAD 1983 State Plane North Carolina FIPS 3200 Feet.

Figure 5 zooms in to show the local correlation for the three MSAs previously presented: Los Angeles-Long Beach-Anaheim (Panel A), Kansas City (Panel B), and Raleigh-Cary (Panel C) MSAs. The standard Pearson correlation coefficients calculated for Los Angeles, Kansas City, and Raleigh-Cary are 0.25, 0.44, and 0.58, respectively. Figure 5 demonstrates within-MSA heterogeneity in the local RI-EI correlation, as well as between-MSA variability in patterning of the local RI-EI correlation. For example, in the Kansas City MSA, there are a few census tracts with negative correlations between RI and EI (shown in blue) as well as a distinctive pattern of high RI-EI correlations in several clusters in the urban center. In contrast, in the Raleigh-Cary MSA, the spatial patterning in local correlation is less distinctive, and there are no tracts with negative RI-EI correlations and few tracts with correlations near 0.

**Figure 6:**
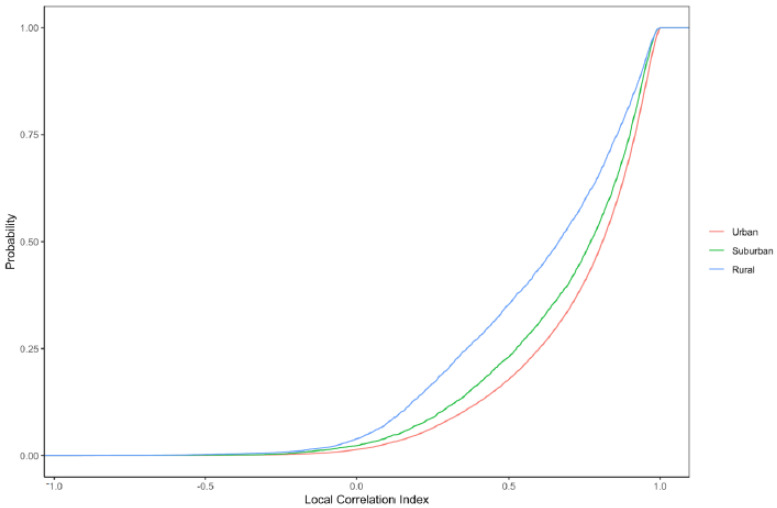
Cumulative distribution functions of the local correlation coefficient between RI and EI in urban, suburban, and rural census tracts.

Lastly, we examine the cumulative distribution functions of local correlation coefficients across tracts with differing levels of urbanicity (Figure 6). The shape of the distribution differs somewhat for different urbanicity categories, with lower correlations (e.g., <0.50) more common in rural tracts and high correlations (e.g., >0.90) more common in urban tracts.

### 3.3. Sensitivity Analysis

The values of the local correlation index $R_{i}$ (Equation (4)) will vary depending on the weights that are assigned for index tract vs. first and second-order neighbors to the index tract. Intuitively, as described in the main analysis, index tracts were assigned the greatest weight ($w_{ii}$ = 1.5), followed by first-order ($w_{ij}$ = 1.0) and then second-order *(*$w_{ij}$ = 0.5) neighbors. To better understand the sensitivity of $R_{i}$ to choice of weights, we explored several different weighting schemes. The local correlation ($R_{i}$) exhibited some sensitivity to choice of weighting scheme. For example, if the index tract was given greater weight ($w_{ii}$ = 5, instead of $w_{ii}$ = 1.5), the mean $R_{i}$ was 0.95 (compared to 0.71 in the main analysis). When first-order neighbors were weighted more heavily ($w_{ij}$ = 1.5, instead of $w_{ij}$ = 1.0), the mean $R_{i}$ changed relatively little (0.72 vs. 0.71). When second-order neighbors were weighted more heavily, ($w_{ij}$ = 1.0, instead of $w_{ij}$ = 0.5), the mean $R_{i}$ was much lower (0.27). Different weights for the index tract, first-order neighboring tracts, and second-order neighboring tracts and resulting mean $R_{i}$ values are presented in Tables S2, S3, and S4, respectively.

### 3.4. Public Health Relevance: RI, EI, and Pregnancy Outcomes

Summary statistics of the analysis births dataset (*n* = 807,991) are provided in Table 1. The analysis dataset included approximately 20% NHB women and 80% NHW women. Mean gestational age was slightly shorter for NHB women (38.1 weeks) compared to NHW women (38.6 weeks). Over 14% of births to NHB women occurred prior to 37 weeks’ gestation compared to less than 10% of births to NHW women. NHB women were less likely than NHW women to report smoking during pregnancy (15.9% vs. 20.1%, respectively). On average, NHB women were younger and had lower educational attainment than NHW women at time of birth. Approximately 25% of NHB women did not graduate from high school compared to 11% of NHW women. Fewer than 10% of NHB women had a college degree compared to 32% of NHW women.

The maternal age distribution in the initial births dataset was similar but not identical to that of the analysis dataset (SM, Table S5). The initial dataset had a higher proportion of mothers that were not high school graduates (16.4%) compared to the analysis dataset (13.9%). A lower proportion of mothers reported smoking in the initial dataset (16.9%) compared to the analysis dataset (19.3%). The proportions of births that were male and preterm were similar in the analysis and initial datasets, as were average census tract-level RI and EI. 

Gestational age in weeks and PTB were examined separately in race-stratified linear and logistic regression models, respectively. Among women of both races, higher tract-level RI and EI were associated with shorter gestation length in weeks (Table 2). Specifically, moving from the median of the lowest (first) quintile of RI to the median of the highest quintile of RI (equivalent to a 0.61 increase in RI) was associated with decrements of −0.069 (95% CI: −0.10, −0.034) and −0.10 (−0.14, −0.065) in gestational length among NHB and NHW women, respectively. Moving from the lowest EI quintile to the highest EI quintile was associated with decrements of −0.066 (−0.12, −0.047) and −0.067 (−0.087, −0.018) in gestational length among NHB and NHW women, respectively. Full results from this model are provided in SM Table S6.

Cross-sectional associations were also observed between RI, EI, and preterm birth (Table 3). Among NHB women, moving from the lowest to the highest quintile of RI was associated with a 1.11 (1.07, 1.15) increase in odds of PTB. Among NHW women, moving from the lowest to the highest RI quintile was also associated with elevated odds of PTB: 1.16 (1.10, 1.22). Among NHB women, the cross-sectional association for moving from the lowest quintile of EI to the highest quintile of EI was 1.07 (1.02, 1.12); for NHW women, it was 1.03 (1.00, 1.05). Full results from this model are provided in SM Table S7.

In a sensitivity analysis, we fit models that included percentage NHB instead of RI and percentage non-college educated instead of EI. Associations between percentage NHB and pregnancy outcomes were generally similar to those reported for RI and pregnancy outcomes; that is, the associations between RI and pregnancy outcomes were not identical to those for percentage NHB and pregnancy outcomes, but associations were not statistically different from one another. Additionally, all associations that were statistically significant between RI and pregnancy outcomes were also statistically significant for percentage NHB and pregnancy outcomes among both NHB and NHW mothers. 

Associations between percentage non-college educated and pregnancy outcomes were also not statistically different from associations between EI and pregnancy outcomes. However, not all associations that were statistically significant for EI–pregnancy outcome were significant for percentage non-college educated and pregnancy outcome. Namely, among NHB mothers, there was a statistically significant, negative association between EI and gestational age as well as between EI and PTB. However, the associations among NHB mothers between percentage non-college educated, gestational age, and PTB were negative but not statistically significant (SM, Tables S8 and S9). 

It is important to recognize that differences in the magnitude, direction, and/or statistical significance of associations for RI vs. percentage NHB or EI vs. percentage not college educated will depend on a number of factors. These include, but are not limited to, the study area, population, outcome of interest, and geographic resolution at which RI (or percentage NHB) and EI (or percentage non-college educated) are calculated (e.g., tract vs. block group vs. county). Thus, this sensitivity analysis is intended to be illustrative, not definitive.

## 4. Discussion

In the US, people are increasingly being spatially sorted by where they live and with whom they interact. A by-product of increasing inequality and the shrinking of the middle class, this phenomenon has profound consequences for opportunities and choices, especially so for our most vulnerable populations. Richard Rothstein has written eloquently about how policies have relentlessly promoted this spatial sorting in *The Color of Law* [48].

A burgeoning literature has highlighted the staggering growth and deleterious consequences of individuals and subpopulations becoming geographically and socially marginalized. *Evicted*, *Our Kids*, and *$2.00 a Day*, as well as a growing body of scholarship by Raj Chetty et al., all underscore the fact that the American Dream of upward mobility is becoming out of reach for many in our country [39,49,50,51]. Systematic measures of residential segregation over space and time, for both urban and non-urban areas, provide a critical tool for assessing the impact of past policy interventions and for identifying new policy approaches.

Our objective was to develop parallel racial and educational isolation indices to serve as tools for health disparities researchers and for those developing intervention programs. Traditional wisdom assumes that race and socioeconomic status are highly correlated. In fact, the global correlation between racial and educational isolation was 0.21. However, we show that this global measure masks local heterogeneity in EI and RI correlation. The pattern of racial and educational isolation differs dramatically across the geography of the US, with notable differences among urban, suburban, and rural areas. At low levels of RI, we observed a relatively wide range of corresponding EI values, but at high levels of RI, the range of EI values narrows to predominantly high EI values. Thus, residents who reside in racially isolated communities are very likely to also experience educational isolation. Additionally, although we have only demonstrated this descriptively in maps, the south is unique in having high RI values in rural settings; elsewhere in the country, high RI values primarily or exclusively occur in urban and suburban areas. Thus, residents of suburban and rural tracts in the South are more likely to experience a “double” exposure to high RI and high EI, compared to other regions of the US, in which suburban and rural tracts may have high EI values but typically have low RI values. 

The distributions and spatial patterns of RI and EI differ from one another. We purposefully calculated RI and EI such that high values of each index indicated greater adversity: high levels of RI indicate greater segregation of NHB relative to the rest of the population, and high levels of EI indicate greater segregation of non-college educated individuals from college educated individuals. However, RI distributions across all levels of urbanicity were right skewed, with more places having low values of RI, while EI was left skewed, with more places tending to have high values of EI. Based on the local correlation coefficient between RI and EI, we find that the coefficients are positive in most census tracts in the continental US. Importantly, values of EI and RI can be highly correlated if EI and RI are both high in an area (e.g., in the southeastern crescent apparent in Figure 1A,B, as well as Figure 4) or because EI and RI are both low in an area (e.g., in parts of the intermountain west).

A local, spatial measure of residential segregation has several advantages over global, aspatial measures. First, residential segregation is an inherently spatial phenomenon, and, therefore, aspatial measures of residential segregation pose methodological concerns, with notable issues related to neighboring communities (i.e., “checkboard problem”) [23]. Since surrounding communities can influence an individual’s neighborhood, a spatial approach enhances the capability of estimating the actual environment where a person lives. This is especially important when considering individuals who reside near bordering geographies.

Second, a local scale is important to understanding residential segregation because many resources are distributed and accessed at a community level [52]. Indeed, many mechanisms of residential segregation, such as school districts, grocery store access, social networks, and air pollution, are geographically constrained. A local measure of residential segregation allows researchers to tap into more proximal environmental influences, whereas the spatial component simultaneously considers the influence of the surrounding environment. Taken together, a local, spatial measure of residential segregation captures the spatial structure of residential segregation at a more relevant and more resolved scale compared to global, aspatial residential segregation measures.

Third, implementing a local, spatial measure at the census tract level, a highly resolved spatial scale, enabled us to distinguish between urban and non-urban census tracts and assess residential segregation in both urban and non-urban areas. There is often an urban bias in segregation and health research, resulting from the predominant focus on MSAs [10]. Although urban areas are important to residential segregation research, segregation also occurs within non-urban areas [10] and has been linked to environmental health risks, regardless of urbanicity [4,6,7,53]. Indices that can measure residential segregation in various regions, including non-urban areas, are a valuable, albeit under-researched, component of residential segregation research.

Finally, a local, spatial measure of residential segregation can also be calculated at or aggregated to different geographic scales, capturing residential segregation at different scales. Depending on the research questions and variables of interest, different geographic scales may be more relevant. Data availability may be a constraint in some cases. For example, the block level is the most highly resolved census geography and serves as the “building blocks” for all other census geographies. Racial composition data are available at the block level, but educational attainment composition data are not, due to privacy considerations. 

To illustrate the utility and relevance of EI and RI, we used eight years of detailed birth records in Michigan to estimate associations with gestation length and preterm birth, which is a leading cause of infant mortality and morbidity that in the US is characterized by stark racial/ethnic disparities. We observed statistically significant associations between RI, EI, and birth outcomes. Specifically, both RI and EI were associated with decrements in gestational length among NHB and NHW women. RI was associated with PTB among NHB and NHW women, while EI was associated with PTB among NHB women only.

Several points merit discussion. First, racial and educational isolation have adverse effects for both NHB and NHW women. Second, the vast majority of NHB women give birth in highly racially isolated neighborhoods. Third, the patterns of co-exposure to RI and EI differ by race/ethnicity. Compared to NHW women, NHB women in Michigan tended to live in neighborhoods that are both racially and educationally isolated, indicating that NHB women are more likely to be co-exposed to EI and RI. Fourth, although the cross-sectional associations reported here are not large in magnitude, these are population-level estimates that apply to a large number of people and therefore translate into substantial public health effects for more racially and educationally isolated populations. 

This paper has several limitations. We dichotomized EI to calculate the isolation of non-college educated from college educated individuals, but there are of course other possible groupings (e.g., non-high school educated vs. high school educated). We also recognize the Modifiable Areal Unit Problem, which introduces bias resulting from the aggregation of point-based measures of spatial phenomena into areal units [54]. In short, the summary values produced from areal/zonal aggregation are arbitrarily determined by both the size and geometry of the aggregation unit [55]. 

Furthermore, in calculating spatial indices, edge effects may occur when neighboring spatial units, in this case census tracts, located outside the study area are ignored, thus distorting the index values assigned to bordering tracts within the study area. For this reason, we calculated RI and EI for the entire US, ignoring state borders. Nonetheless, there is still potential for “edge effects” along coastlines and international borders with Canada and Mexico. Values and patterns of local correlation coefficients may differ at finer or coarser spatial scales, and they also differ depending on how index and neighboring tracts are weighted in the calculation of the local correlation index. Furthermore, census-defined areal units may not accurately represent any given individual’s neighborhood boundaries. The first-order adjacency structure may not accurately represent an individual’s residential environment. Results may be sensitive to our choice regarding the weighting of first-order neighbors in the calculation of EI and RI, and first and second-order neighbors in the calculation of local correlation between EI and RI. The weights are set globally, even though it might be more appropriate to change weights based on local context. These novel local measures can be calculated at varying spatial resolutions, and results could be evaluated for sensitivity regarding the selection of weights. Finally, there are multiple approaches to assessing segregation [16], and no single measure is perfect. Thus, future work to understand how segregation measures relate to one another, as well socioeconomic characteristics, is warranted. 

## 5. Conclusions

Racial residential segregation, and racial isolation as a measure of segregation, has been linked to health [1,4,6,7,8]; although the measure of EI that we introduce here is new, educational attainment is associated with poverty, employment, and income, all of which are unequivocally linked with health [56,57]. Increasingly, the United States is segregated by income [58] and race [11]. A premise of this work is that place-based exposures, including EI and RI, may underlie health and health disparities, and should both be considered and evaluated as possible adverse exposures. A key takeaway of this research is that RI and EI do not exhibit a uniform or random relationship across the United States; rather, we see differences in the relationship between RI and EI across geographic space and urbanicity levels. Specifically, in the south, we observe census tracts that are educationally isolated, racially isolated, and non-urban; elsewhere in the US, tracts that are educationally and racially isolated are found only in urban areas. For example, the health disparities and poor health outcomes that have been documented in the “Stroke Belt” [59] could relate to multiple-jeopardy situations in which adverse exposures (racial isolation, educational isolation, rurality, pollution, access to health care, among others) accumulate to specific populations, by virtue of exposures sustained, in part, due to where they live.

The RI and EI indices described here are available (https://www.cehidatahub.org/hub) and immediately useful to both public health researchers and to policymakers. For researchers, initial work has already linked RI to health outcomes. Further work, including examining the impact of RI and EI separately and jointly, and whether their impact differs by race or geography, is warranted. The local correlation measure developed here can be used to evaluate relationships between other widely used socioeconomic variables measured at various spatial resolutions, which overcomes the tendency of the standardly used global correlation coefficient to mask substantial local heterogeneity in correlation patterns.

In addition, information on RI and EI can help policymakers identify potentially disadvantaged communities and the nature of the disadvantage. Specifically, different intervention strategies may be more appropriate and/or effective depending on whether a community is racially isolated versus educationally isolated, or both. For example, in communities in which few people have a college degree, community leaders may want to first understand whether youth in these communities are not pursuing college degrees or whether they are pursuing and receiving a college education and choosing not to return to the community. Depending on the answer, in such communities, designing policies or programs that identify barriers to college education, emphasize the benefits of college education, or help connect students with mentors or financial resources may be important steps. 

For policymakers, to the extent that RI and EI are linked to health disparities, policy choices can be evaluated for their impact on RI and EI. This is more straightforward than linking to health outcomes directly and thus may get critical information into the policy process more quickly. We note finally that these measures of isolation are not meant to be causal in and of themselves but rather represent the likely accumulation of dis-amenities and overall disinvestment in communities that are racially and educationally isolated. Thus, patterns of RI and EI over time may also represent a way to assess the overall health of a community or specific areas that warrant additional attention or investment. Finally, we argue for shifting the conversation from *race* (non-modifiable) as a driver of outcomes to the *experience and exposures of minorities in segregated communities* (modifiable) as a driver of outcomes. 

## Figures and Tables

**Table 1:** Summary statistics of the Michigan births analysis dataset ^a^.

	Non-Hispanic Black Women (n = 166,720)	Non-Hispanic White Women (n = 641,271)
Neighborhood characteristics		
RI of non-Hispanic Blacks, mean (SD)	0.55 (0.33)	0.074 (0.11)
EI of non-college educated individuals, mean (SD)	0.82 (0.12)	0.75 (0.13)
Infant characteristics		
Gestational age, weeks, mean (SD)	38.1 (2.88)	38.6 (2.10)
Preterm birth	24,070 (14.4)	59,847 (9.33)
Male	85,015 (50.9)	328,712 (51.2)
Maternal characteristics ^b^		
Reported smoking during pregnancy (1 = smoker)	26,551 (15.9)	129,098 (20.1)
Unmarried at time of birth (1 = unmarried)	131,944 (79.0)	194,893 (30.4)
Age at birth (years)		
15–19	31,195 (18.7)	42,781 (6.67)
20–24	54,972 (32.9)	142,758 (22.2)
25–29	39,093 (23.4)	200,355 (31.2)
30–34	25,713 (15.9)	166,941 (26.0)
35–39	12,898 (77.2)	72,960 (11.4)
40–44	2849 (1.71)	15,476 (2.41)
Educational attainment		
Less than high school	42,913 (25.7)	69,586 (10.8)
High school diploma	107,895 (64.6)	363,572 (56.7)
College diploma or higher	15,912 (9.53)	208,113 (32.4)

^a^ The cell count and percent are presented except in the case of RI, EI, and gestation length, for which the mean and standard deviation are given as indicated next to the variable name. ^b^ Maternal variables are based on reported maternal characteristics at time of the neonate’s birth.

**Table 2:** Associations between gestational age in weeks and racial isolation and educational isolation ^a^.

	NH Black	NH White
RI of non-Hispanic Blacks	−0.069 (−0.10, −0.034)	−0.10 (−0.14, −0.065)
EI of non-college educated individuals	−0.066 (−0.12, −0.047)	−0.067 (−0.087, −0.018)

^a^ Models adjusted for individual-level characteristics, including maternal age at time of giving birth (15–19, 20–24, 25–29, 30–34, 35–40, or 40–44 years), maternal educational attainment (not a high school graduate; high school graduate; or college graduate), maternal marital status at time of birth (married or unmarried), maternal tobacco use during pregnancy, and infant sex. The census tract of maternal residence at time of birth was included as a random effect.

**Table 3:** Associations between preterm birth and racial isolation and educational isolation ^a^.

	Odds Ratios (95% Confidence Interval)	
	NH Black	NH White
RI of non-Hispanic Blacks	1.11 (1.07, 1.15)	1.16 (1.10, 1.22)
EI of non-college educated individuals	1.07 (1.02, 1.12)	1.03 (1.00, 1.05)

^a^ Models adjusted for individual-level characteristics, including maternal age at time of giving birth (15–19, 20–24, 25–29, 30–34, 35–40, or 40–44 years), maternal educational attainment (not a high school graduate; high school graduate; or college graduate), maternal marital status at time of birth (married or unmarried), maternal tobacco use during pregnancy, and infant sex. The census tract of maternal residence at time of birth was included as a random effect.

## Data Availability

Datasets are publicly available with the exception of the Michigan detailed birth records, which is governed by an IRB at the University of Notre Dame and the Michigan Department of Health and Human Services Children’s Environmental Health Initiative (2020). Racial isolation values based on the 2010 census tract level using the 2010 census boundaries (dataset). CEHI. https://doi.org/10.25614/ri_us_2010_trt. Children’s Environmental Health Initiative (2020). Educational isolation values based on the 2010 census tract level using the 2008–2012 ACS 5-year population estimates (dataset). https://doi.org/10.25614/ei_us_2010_trt.

## Supplementary Materials

The following are available online at https://www.mdpi.com/article/10.3390/ijerph18179384/s1, Table S1: Urbanicity classification based on Rural–Urban Commuting Area (RUCA) codes; Table S2: Summary statistics of initial and analysis dataset of Michigan births; Table S3: Associations between gestational age in weeks and racial isolation and educational isolation; Table S4: Associations between preterm birth and racial isolation and educational isolation; Table S5: Sensitivity Analysis of $R_{i}$ for different $w_{ii}$; Table S6: Sensitivity Analysis of $R_{i}$ for different $w_{ij}$ of first order neighbors; Table S7: Sensitivity Analysis of $R_{i}$ for different $w_{ij}$ of second order neighbors; Table S8: Comparison of associations between gestational age in weeks and racial isolation vs % NHB and educational isolation vs % non-college educated; Table S9: Comparison of odds ratios between preterm birth and racial isolation vs % NHB and educational isolation vs % non-college educated.

## References

[1] Williams D.R., Collins C. (2001). Racial residential segregation: A fundamental cause of racial disparities in health. Public Health Rep..

[2] Jackson S.A., Anderson R.T., Johnson N.J., Sorlie P.D. (2000). The Relation of Residential Segregation to All-Cause Mortality: A Study in Black and White. Am. J. Public Health.

[3] Polednak A.P. (1996). Trends in US urban black infant mortality, by degree of residential segregation. Am. J. Public Health.

[4] Anthopolos R., James S.A., Gelfand A.E., Miranda M.L. (2011). A spatial measure of neighborhood level racial isolation applied to low birthweight, preterm birth, and birthweight in North Carolina. Spat. Spatiotemporal. Epidemiol..

[5] Britton M.L., Shin H. (2013). Metropolitan residential segregation and very preterm birth among African American and Mexican origin women. Soc. Sci. Med..

[6] Bravo M.A., Anthopolos R., Kimbro R.T., Miranda M.L. (2018). Residential Racial Isolation and Spatial Patterning of Type 2 Diabetes Mellitus in Durham, North Carolina. Am. J. Epidemiol..

[7] Bravo M.A., Batch B., Miranda M.L. (2019). Residential racial isolation and spatial patterning of hypertension in Durham, North Carolina. Prev. Chronic Dis..

[8] Kershaw K.N., Robinson W.R., Gordon-Larsen P., Hicken M.T., Goff D.C.J., Carnethon M.R., Kiefe C.I., Sidney S., Diez Roux A.V. (2017). Association of Changes in Neighborhood-Level Racial Residential Segregation with Changes in Blood Pressure Among Black Adults: The CARDIA Study. J. Am. Med Assoc. Intern. Med..

[9] Kershaw K.N., Albrecht S.S. (2015). Racial/ethnic residential segregation and cardiovascular disease risk. Curr. Cardiovasc. Risk Rep..

[10] Lichter D.T., Parisi D., Grice S.M., Tacquino M.C. (2007). National estimates of racial segregation in rural and small-town America. Demography.

[11] Massey D.S., Denton N.A. (1988). The Dimensions of Residential Segregation. Soc. Forces.

[12] Hearst M.O., Oakes J.M., Johnson P.J. (2008). The effect of racial residential segregation on black infant mortality. Am. J. Epidemiol..

[13] Morello-Frosch R., Lopez R. (2006). The Riskscape and the Colorline: Examining the Role of Segregation in Environmental health Disparities. Environ. Res..

[14] Jones M.R., Diez-Roux A.V., Hajat A., Kershaw K.N., O’Neill M.S., Guallar E., Post W.S., Kaufman J.D., Navas-Acien A. (2014). Race/ethnicity, residential segregation, and exposure to ambient air pollution: The multi-ethnic study of atherosclerosis (MESA). Am. J. Public Health.

[15] Logan T.D., Parman J.M. (2017). The national rise in residential segregation. J. Econ. Hist..

[16] White K., Borrell L.N. (2011). Racial/ethnic residential segregation: Framing the context of health risk and health disparities. Health Place.

[17] Acevedo-Garcia D., Lochner K., Osypuk T.L., Subramnian S.V. (2003). Future directions in residential segregation and health research: A multilevel approach. Am. J. Public Health.

[18] Massey D.S., Denton N.A. (1993). American Apartheid: Segregation and the Making of the Underclass.

[19] Massey D.S., Denton N.A. (1989). Hypersegregation in U.S. metropolitan areas: Black and Hispanic segregation along five dimensions. Demography.

[20] Lee B.A., Reardon S.F., Firebaugh G., Farrell C.R., Matthews S.A., O’Sullivan D. (2008). Beyond the Census Tract: Patterns and Determinants of Racial Segregation at Multiple Geographic Scales. Am. Sociol. Rev..

[21] Quillian L. (2012). Segregation and Poverty Concentration: The Role of Three Segregations. Am. Sociol. Rev..

[22] Orfield G., Eaton S.E. (1996). Dismantling Desegregation. The Quiet Reversal of Brown v. Board of Education.

[23] White M.J. (1983). The measurement of spatial segregation. Am. J. Sociol..

[24] Reardon S.F., Farrell C.R., Matthews S.A., O’Sullivan D., Bischoff K., Firebaugh G. (2009). Race and space in the 1990s: Changes in the geographic scale of racial residential segregation, 1990–2000. Soc. Sci. Res..

[25] Reardon S.F., O’Sullivan D. (2004). Measures of spatial segregation. Sociol. Methodol..

[26] Shihadeh E.S., Flynn N. (1996). Segregation and crime: The effect of black social isolation on the rates of black urban violence. Soc. Forces.

[27] Cohen A.K., Syme S.L. (2013). Education: A missed opportunity for public health intervention. Am. J. Public Health.

[28] Cutler D., Lleras-Muney A. (2006). Education and Health: Evaluating Theories and Evidence. Natl. Bur. Econ. Res. Work. Pap..

[29] Goesling B. (2007). The rising significance of education for health?. Soc. Forces.

[30] Ross C.E., Wu C.L. (1995). The links between education and health. Am. Sociol. Rev..

[31] Woolf S.H., Johnson R.E., Phillips R.L., Philipsen M.A.J.P.H. (2007). Giving everyone the health of the educated: An examination of whether social change would save more lives than medical advances. Am. J. Public Health.

[32] Yen I.H., Moss N. (1999). Unbundling education: A critical discussion of what education confers and how it lowers risk for disease and death. Ann. N. Y. Acad. Sci..

[33] Marcotte D.E., Dalane K. (2019). Socioeconomic segregation and school choice in American public schools. Educ. Res..

[34] Frankel D.M., Volij O. (2011). Measuring school segregation. J. Econ. Theory.

[35] García E. (2020). Schools Are Still Segregated, and Black Children Are Paying a Price.

[36] Saporito S., Sohoni D. (2006). Coloring outside the lines: Racial segregation in public schools and their attendance boundaries. Sociol. Educ..

[37] Owens A., Reardon S.F., Jencks C. (2016). Income segregation between schools and school districts. Am. Educ. Res. J..

[38] Reardon S.F., Weathers E.S., Fahle E.M., Jang H., Kalogrides D. (2019). Is Separate Still Unequal? New Evidence on School Segregation and Racial Academic Achievement Gaps.

[39] Putnam R.D. (2015). Our Kids: The American Dream in Crisis.

[40] LaVeist T., Pollack K., Thorpe R., Fesahazion R., Gaskin D. (2011). Place, Not Race: Disparities Dissipate in Southwest Baltimore When Blacks and Whites Live Under Similar Conditions. Health Aff..

[41] LaVeist T.A., Thorpe R.J., Galarraga J.E., Bower K.M., Gary-Webb T.L. (2009). Environmental and socio-economic factors as contributors to racial disparities in diabetes prevalence. J. Gen. Intern. Med..

[42] United States Census Bureau (2010). 2010 Decennial Census.

[43] United States Department of Agriculture (2010). Rural-Urban Commuting Area Codes.

[44] Children’s Environmental Health Initiative (2020). Racial Isolation Values based on the 2010 Census Tract Level Using the 2010 Census Boundaries.

[45] Children’s Environmental Health Initiative (2020). Educational Isolation Values based on the 2010 Census Tract Level Using the 2008–2012 ACS 5-Year Population Estimates.

[46] Misra T. (2016). A Complex Portrait of Rural America.

[47] Fain P. Race, Geography and Degree Attainment. Inside Higher Ed. https://www.insidehighered.com/news/2019/06/27/rural-areas-lag-degree-attainment-while-urban-areas-feature-big-racial-gaps.

[48] Rothstein R. (2017). The Color of Law: A Forgotten History of How Our Government Segregated America.

[49] Chetty R., Friedman J., Hendren N. (2017). The Equality of Opportunity Project.

[50] Desmond M. (2016). Evicted: Poverty and Profit in the American City.

[51] Edin K.J., Shaefer H.L. (2015). $2.00 a Day: Living on Almost Nothing in America.

[52] Cornwell E.Y., Cornwell B. (2008). Access to expertise as a form of social capital: An examination of race-and class-based disparities in network ties to experts. Sociol. Perspect..

[53] Bravo M.A., Anthopolos R., Bell M.L., Miranda M.L. (2016). Racial isolation and exposure to airborne particulate matter and ozone in understudied US populations: Environmental justice applications of downscaled numerical model output. Env. Int..

[54] Openshaw S. (1984). The Modifiable Areal Unit Problem Concepts and Techniques in Modern Geography 38.

[55] Wong D., Fotheringham A., Rogerson P. (2009). The modifiable areal unit problem (MAUP). The SAGE Handbook of Spatial Analysis.

[56] Chetty R., Stepner M., Abraham S., Lin S., Scuderi B., Turner N., Bergeron A., Cutler D. (2016). The association between income and life expectancy in the United States, 2001–2014. JAMA.

[57] Khullar D., Chokshi D.A. (2018). Health, income, & poverty: Where we are & what could help. Health Aff. Health Policy Brief.

[58] Taylor P., Fry R.A. (2012). The Rise of Residential Segregation by Income.

[59] Wing S., Casper M., Davis W.B., Pellom A., Riggan W., Tyroler H. (1988). Stroke mortality maps. United States whites aged 35–74 years, 1962–1982. Stroke.

