# Supplementary material for: Assessing Disparity Using Measures of Racial and Educational Isolation
Source: Int J Environ Res Public Health. 2021 Sep 6;18(17):9384. doi: 10.3390/ijerph18179384 (PMC8430965; doi:10.3390/ijerph18179384)
Supplement: Supplementary file 1 [file ijerph-18-09384-s001.zip › ijerph-1284055-supplementary.pdf]

SUPPLEMENTAL MATERIAL

Assessing disparity using measures of racial and educational isolation

Mercedes A. Bravo [1,2], Man Chong Leong[2,3], Alan E. Gelfand[4] and Marie Lynn Miranda[2,5,*]

[1] Global Health Institute, Duke University, Durham, NC, USDA
[2] Children's Environmental Health Initiative, University of Notre Dame, IN, USA
[3] Department of Biostatistics, University of Florida, Gainesville, FL, USA
[4] Department of Statistical Science, Duke University, Durham, NC, USA
[5] Department of Applied and Computational Mathematics and Statistics, University of Notre Dame, IN, USA
* Correspondence: mlm@nd.edu

SM Table S1. Urbanicity classification based on Rural-Urban Commuting Area (RUCA) codes

Classification	Primary codes	Secondary codes
Urban	1.0 Metropolitan area core: primary flow within an urbanized area (UA)<br><br>2.0 Metropolitan area high commuting: primary flow 30% or more to a UA<br><br>3.0 Metropolitan area low commuting: primary flow 10% to 30% to a UA	1.1 Secondary flow 30% to 50% to a larger UA<br><br>2.1 Secondary flow 30% to 50% to a larger UA<br><br>4.1 Secondary flow 30% to 50% to a UA<br><br>5.1 Secondary flow 30% to 50% to a UA<br><br>7.1 Secondary flow 30% to 50% to a UA<br><br>8.1 Secondary flow 30% to 50% to a UA<br><br>10.1 Secondary flow 30% to 50% to a UA
Suburban	4.0 Micropolitan area core: primary flow within an urban cluster of 10,000 to 49,999 (large UC)<br><br>5.0 Micropolitan high commuting: primary flow 30% or more to a large UC<br><br>6.0 Micropolitan low commuting: primary flow 10% to 30% to a large UC	

Rural	7.0 Small town core: primary flow within an urban cluster of 2,500 to 9,999 (small UC)<br><br>8.0 Small town high commuting: primary flow 30% or more to a small UC<br><br>9.0 Small town low commuting: primary flow 10% to 30% to a small UC<br><br>10.0 Rural areas: primary flow to a tract outside a UA or UC	7.2 Secondary flow 30% to 50% to a UA<br><br>8.2 Secondary flow 30% to 50% to a large UC<br><br>10.2 Secondary flow 30% to 50% to a large UC<br><br>10.3 Secondary flow 30% to 50% to a small UC

SM Table S2. Sensitivity Analysis of R_i for different w_{ii}.

w_{ii} weight	w_{ij} weight – first order neighbor	w_{ij} weight – second order neighbor	Mean (Median) of R_i
1.0	1.0	0.5	0.701 (0.761)
**1.5**	**1.0**	**0.5**	**0.705 (0.793)**
3.0	1.0	0.5	0.862 (0.963)
5.0	1.0	0.5	0.951 (0.994)
10.0	1.0	0.5	0.991 (0.999)
50.0	1.0	0.5	0.998 (1)
100.0	1.0	0.5	0.999 (1)

The row of values highlighted in bold correspond to the weighting scheme presented in the main analysis (w_{ii} = 1.5 for the index tract).

SM Table S3. Sensitivity Analysis of R_i for different w_{ij} of first order neighbors.

w_{ii} weight	w_{ij} weight – first order neighbor	w_{ij} weight – second order neighbor	Mean (Median) of R_i
1.5	0.5	0.5	0.845 (0.908)
1.5	0.75	0.5	0.786 (0.860)
**1.5**	**1.0**	**0.5**	0.705 (0.793)
1.5	1.25	0.5	0.683 (0.773)
1.5	1.50	0.5	0.722 (0.805)

The row of values highlighted in bold correspond to the weighting scheme presented in the main analysis (w_{ij} = 1.0 for first order neighbors).

**SM Table S4.** Sensitivity Analysis of R_i for different w_{ij} of second order neighbors.			
w_{ii} weight	w_{ij} weight – first order neighbor	w_{ij} weight – second order neighbor	Mean (Median) of R_i
1.5	1.0	0.01	0.622 (0.716)
1.5	1.0	0.1	0.756 (0.833)
1.5	1.0	0.25	0.785 (0.759)
**1.5**	**1.0**	**0.5**	**0.705 (0.793)**
1.5	1.0	1.0	0.271 (0.288)

The row of values highlighted in bold correspond to the weighting scheme presented in the main analysis (w_{ij} = 0.5 for second order neighbors).

SM Table S5. Summary statistics of initial and analysis dataset of Michigan births[a]		
	All births data (n=1,608,537)	Analysis data set (n=807,991)
Born between 2005-2012	954,455 (59.3)	807,991 (100.0)
Geocoded	1,583,214 (98.4)	807,991 (100.0)
**Neighborhood characteristics**		
RI of non-Hispanic Blacks, mean (SD)	0.17 (0.26)	0.17 (0.26)
EI of non-college educated individuals, mean (SD)	0.76 (0.14)	0.76 (0.13)
Infant characteristics		
Gestational age, weeks, mean (SD)	38.6 (2.3)	38.5 (2.2)
Preterm birth	164,995 (10.3)	83,304 (10.3)
Male	824,368 (51.2)	413,727 (51.2)
**Maternal characteristics**[b]		
Race/ethnicity		
Non-Hispanic Black	286,722 (17.8)	166,720 (20.6)
Non-Hispanic White	1,121,014 (69.7)	641,271 (79.4)
Hispanic	103,447 (6.43)	0 (0)
Non-Hispanic Asian/Pacific Islander	52,301 (3.25)	0 (0)
Non-Hispanic other	23,633 (1.47)	0 (0)
Reported smoking during pregnancy (1=smoker)	271,111 (16.9)	155,649 (19.3)
Age at birth (years)		
15-19	153,118 (9.52)	73,976 (9.16)
20-24	391,230 (24.3)	197,730 (24.5)
25-29	463,868 (28.8)	239,448 (29.6)
30-34	388,431 (24.1)	192,654 (23.8)
35-39	172,318 (10.7)	85,858 (10.6)
40-44	35,126 (2.18)	18,325 (2.27)
Educational attainment		
Less than high school	263321 (16.4)	112,499 (13.9)
High school diploma	893,630 (55.6)	471,467 (58.3)
College diploma or higher	427,007 (26.5)	224,025 (27.8)
Unmarried at time of birth (1=unmarried)	613,859 (38.2)	326,837 (40.5)

[a]The cell count and percent are presented except in the case of RI, EI, and gestataion length, for which the mean and standard deviation are given as indicated next to the variable name. [b] Maternal variables are based on reported maternal characteristics at time of the neonate's birth.

SM Table S6. Associations between gestational age in weeks and racial isolation and educational isolation		
	**non-Hispanic Black**	**non-Hispanic White**
**Neighborhood characteristics**		
RI of non-Hispanic Blacks	-0.069 (-0.10, -0.034)	-0.10 (-0.14, -0.065)
EI of non-college educated individuals	-0.066 (-0.12, -0.047)	-0.067 (-0.087, -0.018)
**Individual characteristics**		
Male infant	-0.007 (-0.033, 0.020)	-0.076 (-0.086, -0.066)
Reported smoking during pregnancy (1=smoker)	-0.14 (-0.18, -0.10)	-0.10 (-0.12, -0.087)
Age at birth (years)		
15-19	0.32 (0.27, 0.36)	0.15 (0.13, 0.18)
20-24	0.19 (0.15, 0.22)	0.10 (0.087, 0.12)
25-29	Reference	Reference
30-34	-0.18 (-0.23, -0.14)	-0.13 (-0.15, -0.12)
35-39	-0.44 (-0.50, -0.39)	-0.28 (-0.30, 0.26)
40-44	-0.46 (-0.56, -0.35)	-0.38 (-0.42, -0.35)
Educational attainment		
Less than high school	-0.040 (-0.074, -0.0071)	-0.079 (-0.097, -0.061)
High school diploma	Reference	Reference
College diploma or higher	0.11 (0.055, 0.16)	0.081 (0.069, 0.094)
Unmarried at time of birth (1=unmarried)	-0.12 (-0.15, -0.078)	-0.030 (-0.044, -0.017)

SM Table S7. Associations between preterm birth and racial isolation and educational isolation		
	**Odds ratios (95% confidence intervals)**	
	**non-Hispanic Black**	**non-Hispanic White**
**Neighborhood characteristics**		
RI of non-Hispanic Blacks	1.11 (1.07, 1.15)	1.16 (1.10, 1.22)
EI of non-college educated individuals	1.07 (1.02, 1.12)	1.03 (1.00, 1.05)
**Individual characteristics**		
Male infant	1.02 (0.99, 1.05)	1.11 (1.09, 1.13)
Reported smoking during pregnancy	1.16 (1.12, 1.20)	1.12 (1.09, 1.14)
Age at birth (years)		
15-19	0.79 (0.75, 0.82)	0.92 (0.88, 0.96)
20-24	0.85 (0.82, 0.89)	0.92 (0.90, 0.95)
25-29	Reference	Reference
30-34	1.17 (1.12, 1.23)	1.14 (1.12, 1.17)
35-39	1.47 (1.39, 1.54)	1.35 (1.31, 1.39)
40-44	1.59 (1.44, 1.75)	1.47 (1.39, 1.54)
Educational attainment		
Less than high school	1.09 (1.05, 1.12)	1.11 (1.08, 1.15)
High school diploma	Reference	Reference
College diploma or higher	0.91 (0.87, 0.96)	0.94 (0.93, 0.97)
Unmarried at time of birth (1=unmarried)	1.18 (1.13, 1.23)	1.12 (1.09, 1.14)
